# 

**Published:** 1880-04

**Authors:** 


### Table of Contents.

### ORIGINAL COMMUNICATIONS.

A Thesis on the Action of Remedies. By C. C. Maddox.......... 1
An Epidemic of Jaundice with Some Unusual and Remarkable Symptoms By R. J. Nunn, M.D., Savannah, Ga.................... 6

### REPORT OF SOCIETIES.

Cincinnati Medical Society.................................................. 7
New York Academy of Medicine.......................................... 10
A Case of Entire Obstruction of the Bowels for Three Weeks......... 16
The Medico-Legal Society........ ........................................... 24

### SELECTIONS.

The Germ Theory of Disease.................................................. 29
The Early Treatment of Burns and Scalds.................................. 34
Curious Case of Midwifery.................................................. 38
Treatment of Sub-acute and Chronic Gout.................................. 43
Placenta Prævia.............................................................. 50

### EDITORIAL.

Medical Gathering in New York............................................ 53
The National Board of Health—The Medical Profession to the People of Savannah.............................................................. 55
Meteorological Report for March .......................................... 60

### BIBLIOGRAPHICAL.

Common Mind Troubles and the Secret of a Clear Head...... ......... 61

### EXCERPTA.

A Case of Heart Clot ........................................................ 62
A Word of Caution in the Administration of Acid Medicines........... 63

